# Pulsed sputtering epitaxial growth of *m*-plane InGaN lattice-matched to ZnO

**DOI:** 10.1038/s41598-017-12518-w

**Published:** 2017-10-09

**Authors:** Atsushi Kobayashi, Jitsuo Ohta, Hiroshi Fujioka

**Affiliations:** 1 Institute of Industrial Science, The University of Tokyo, Tokyo, 153-8505 Japan; 2 PRESTO, Japan Science and Technology Agency, Saitama, 332-0012 Japan; 3 ACCEL, Japan Science and Technology Agency, Tokyo, 102-0076 Japan

## Abstract

*m*-Plane GaN and InGaN films were grown on *m*-plane ZnO substrates at ~350 °C by pulsed sputtering deposition. It was found that the critical thickness of the *m*-plane GaN films grown on ZnO lies between 25 and 62 nm, whereas 180-nm-thick *m*-plane In_0.12_Ga_0.88_N can be coherently grown on ZnO substrates, which is explained well by theoretical calculations based on an energy-balance model. The coherently grown *m*-plane InGaN on ZnO exhibited narrow X-ray rocking curves compared with the *m*-plane GaN grown on ZnO. These results demonstrate the benefit of lattice-matched ZnO substrates for epitaxy of high-quality nonpolar InGaN films.

## Introduction

**Figure 1:**
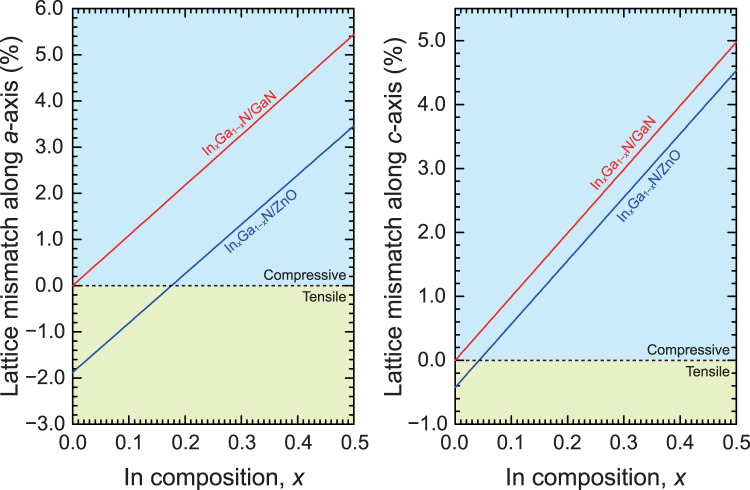
In-plane lattice mismatches of *m*-plane InGaN/ZnO and InGaN/GaN. Lattice constants of InGaN were calculated according to Vegard’s law.

Over the last decade or so, the growth, physics, and device applications of nonpolar and semipolar nitrides have made significant progress^1,2^. The first planar nonpolar *m*-plane GaN was grown on LiAlO_2_ substrates by Waltereit *et al*.^3^, which motivated the researchers to grow nonpolar GaN films on other substrates such as sapphire and SiC for light-emitting diodes (LEDs)^4,5^. Subsequently, the use of nonpolar and semipolar bulk substrates prepared by hydride vapor phase epitaxy (HVPE) increased, enabling the fabrication of bright LEDs and green laser diodes (LDs)^6–9^. Throughout history, it was observed that high-performance devices need to be fabricated on high-quality substrates. Therefore, bulk GaN substrates are now widely used for nonpolar light-emitting devices^10–12^; however, the use of GaN substrates presents the problem of lattice mismatch with InGaN^13,14^. This lattice mismatch between GaN and InGaN could be detrimental, particularly for long-wavelength LEDs and LDs. To overcome the lattice mismatch problem, the use of ZnO substrates has been proposed for the growth of high-quality nonpolar InGaN^15,16^. As shown in Fig. 1, compared with GaN, ZnO exhibits a smaller lattice mismatch along both the *a*- and *c*-axes with In_*x*_Ga_1−*x*_N (*x* > 0.09). Another advantage is that high-quality ZnO substrates can be prepared using hydrothermal methods^17^, and ZnO substrates are larger than the state-of-the-art bulk GaN substrates. It can be noted that the use of ZnO substrates is the best way to fabricate nonpolar InGaN-based devices because nonpolar ZnO substrates are considerably cheaper than nonpolar GaN bulk substrates. It is natural to try to grow nitride films on ZnO because it is the only wurtzite material that is lattice-matched to group III nitrides.

HVPE^18,19^, metalorganic vapor phase epitaxy^20^, and molecular beam epitaxy^21^ of GaN and InGaN on ZnO have been reported, and a reasonable epitaxial relationship of nitride and ZnO has been confirmed^20^. From these results, it can be expected that high-quality nonpolar nitrides could also grow on ZnO substrates. However, it is difficult to grow nitride films on chemically vulnerable ZnO substrates by chemical vapor deposition. To take full advantage of the lattice-matching nature of ZnO and grow high-quality nitride films, the chemical/interfacial reactions between nitrides and ZnO need to be completely suppressed.

A decrease in the growth temperature is important to suppress the interfacial reactions between nitrides and ZnO. Recently, a low-temperature epitaxial growth technique was developed called pulsed sputtering deposition (PSD)^22^. This technique enables the growth of nitride films with high control over the film thickness and allows deposition over a large area. PSD is particularly suitable for InGaN growth because its growth temperature is lower than those of MBE, MOVPE, and HVPE. In PSD growth, a pulsed supply of indium and gallium atoms with high kinetic energy enhances the migration of adatoms on the surface, leading to a dramatic reduction in the growth temperature. Using this technique, high-quality n-type^23^ and p-type^24^ GaN films were successfully grown with room temperature carrier mobilities of 1008 and 34 cm^2^ V^−1^ s^−1^, respectively. Moreover, LEDs were also fabricated at temperatures lower than 500 °C^25^. These results indicate the potential of PSD for the fabrication of nitride-based devices on ZnO substrates. In this article, the structural properties of nonpolar *m*-plane GaN and InGaN films grown on ZnO substrates by PSD are reported.

## Results and Discussion

**Figure 2:**
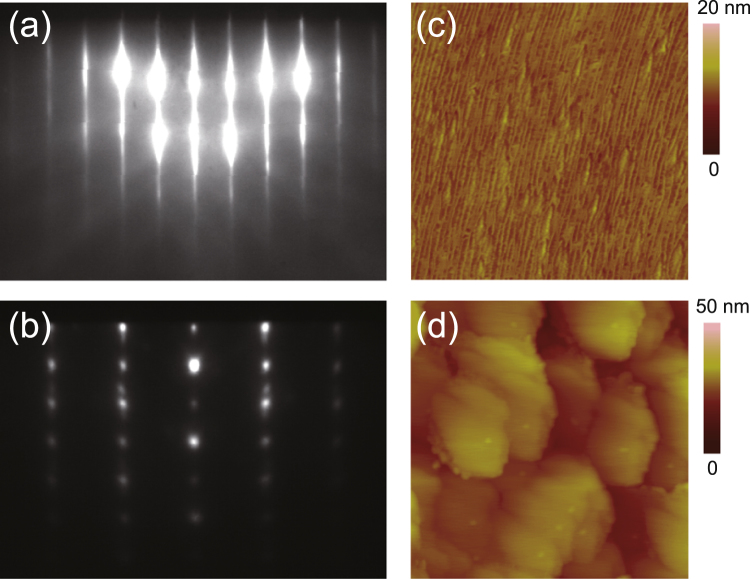
RHEED patterns [(**a**) and (**b**)] and AFM images (1 μm × 1 μm) [(**c**) and (**d**)] of GaN films grown on *m*-plane ZnO substrates at 340 [(**a**) and (**c**)] and 540 °C [(**b**) and (**d**)].

First, the structural properties of the GaN films grown on *m*-plane ZnO were investigated. Figure 2 shows the reflection high-energy electron diffraction (RHEED) patterns [(a) and (b)] and atomic force microscope (AFM) images [(c) and (d)] of GaN films grown at 340 °C [(a) and (c)] and 540 °C [(b) and (d)]. A sharp streaky RHEED pattern corresponding to the growth of *m*-plane GaN was observed for the film grown at 340 °C, whereas the film grown at 540 °C exhibited a RHEED pattern with spots, indicating the growth of a mixture of zincblende and wurtzite GaN. The polycrystalline growth of GaN was also observed on the *m*-plane ZnO substrates when the growth was performed at high temperatures (above 500 °C) using pulsed laser deposition^26^. The failed growth is attributed to the formation of interfacial alloys through the diffusion of Ga and N into ZnO heated above 500 °C. The formation of the interfacial layer results in nitride films with a rough surface, making it difficult to control the electrical and optical properties. AFM observations revealed that the surface of *m*-plane GaN epitaxially grown on ZnO was atomically flat [Fig. 2(c)], which is in striking contrast with the rough surface of GaN grown on ZnO at 540 °C [Fig. 2(d)].

**Figure 3:**
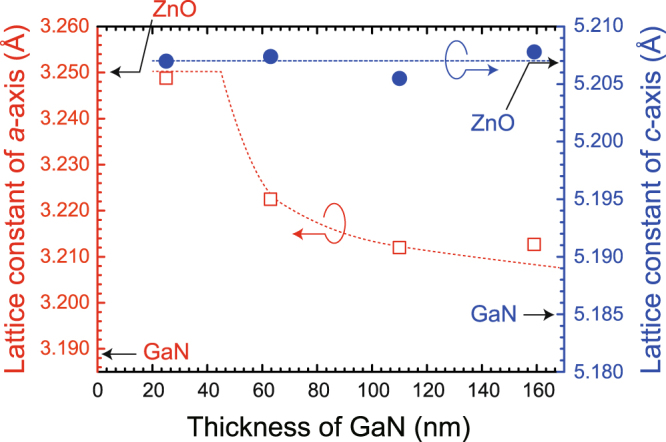
Evolution of lattice constants of GaN films grown on *m*-plane ZnO substrates at 340 °C. Fully relaxed lattice constants of ZnO and GaN are also indicated. Broken lines are guide to the eye.

Next, the structural characterization of a single crystalline *m*-plane GaN film on ZnO was performed using X-ray diffraction (XRD). The full widths at half maximum (FWHM) of the X-ray rocking curves (XRCs) for *m*-plane GaN (25 nm thick) grown at 340 °C were 155 and 130 arcsec with the X-ray incident angle perpendicular to [$11\bar{2}0$] and [0001], respectively. In order to experimentally determine the critical thickness for the lattice relaxation of *m*-plane GaN grown on ZnO by PSD, four samples with different film thicknesses were prepared (25, 62, 110, and 160 nm) and the lattice constants of GaN films were measured using the X-ray reciprocal space map (RSM) technique. Figure 3 shows the lattice constants of GaN films with different thicknesses. While the lattice constant along the *c*-axis was almost unchanged for all film thicknesses, the lattice constant along the *a*-axis relaxed at a film thickness between 25 and 62 nm and gradually approached the lattice constant of the completely relaxed GaN. The predominant lattice relaxation along the *a*-axis can be explained by the larger lattice mismatch of the *a*-axis (1.9%) compared with that of the *c*-axis (0.4%).

**Figure 4:**
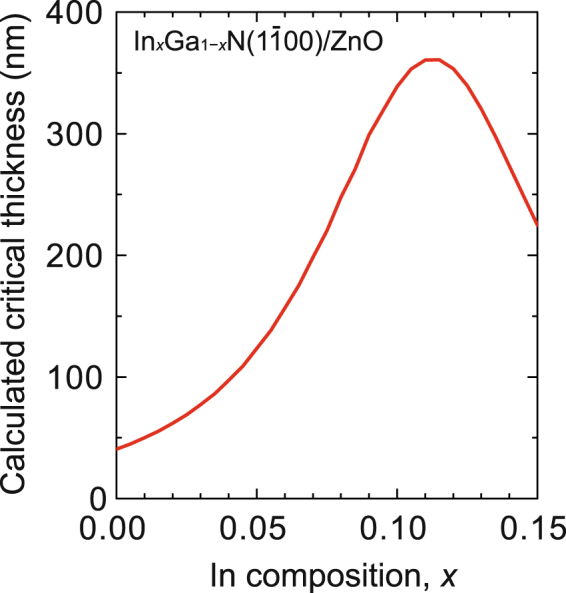
Calculated critical thickness of *m*-plane In_1−*x*_Ga_*x*_N on ZnO. The value reaches a maximum at the In composition of 0.115.

The theoretical critical thicknesses of *m*-plane GaN (and InGaN) *heteroepitaxially* grown on ZnO were calculated by employing the energy-balance model proposed by People and Bean^27^. In this model, the critical thickness is equal to the film thickness at which the energy density of a misfit dislocation coincides with the strain energy density stored in the film. According to Huang *et al*.^28^, the critical thickness *h*_crit_ of nonpolar wurtzite crystals can be expressed as follows:

1 $$\begin{array}{rcl}{h}_{crit} & = & \frac{G{b}^{2}}{16a\pi (1-\nu )U}\,\mathrm{ln}\,\frac{{h}_{crit}}{{r}_{c}}\\ U & = & \frac{{S}_{33}{{\varepsilon }_{xx}}^{2}+{S}_{11}{{\varepsilon }_{zz}}^{2}-2{S}_{13}{\varepsilon }_{xx}{\varepsilon }_{zz}}{2({S}_{11}{S}_{33}-{{S}_{13}}^{2})},\end{array}$$

where *G* is the shear modulus, *b* the magnitude of the Burgers vector of the misfit dislocation, *a* the lattice constant of GaN (or InGaN), *ν* the Poisson’s ratio of GaN, *r*_c_ the core radius of the misfit dislocation, *U* the strain energy per unit volume, *S* the elastic compliance coefficient, and *ε* the strain. Figure 4 depicts the dependence of the calculated critical thickness of *m*-plane In_1−*x*_Ga_*x*_N/ZnO on In composition, *x*. The critical thickness of GaN (*x* = 0) is calculated to be 45 nm, which well explained the experimental result shown in Fig. 3. The critical thickness reaches a maximum value of 360 nm at the In composition of 11.5%. The lattice mismatches between In_0.115_Ga_0.885_N and ZnO are −0.65% (*a*-axis) and + 0.72% (*c*-axis). For an In_0.115_Ga_0.885_N film on ZnO, both the strains are balanced, resulting in the maximum critical thickness.

**Figure 5:**
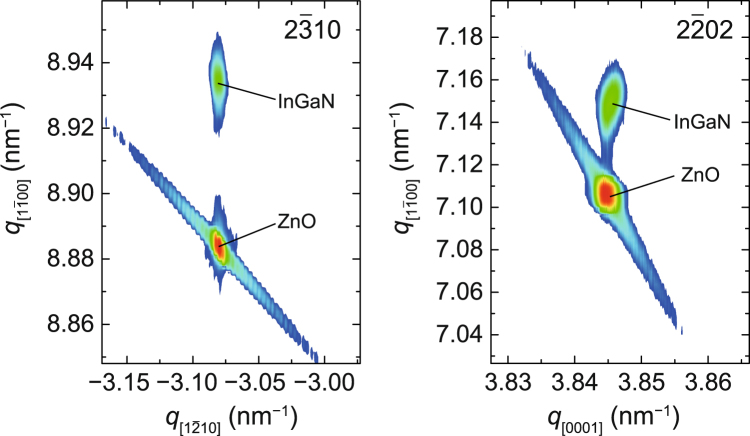
X-ray RSMs of *m*-plane In_0.12_Ga_0.88_N/ZnO around $2\bar{3}10$ (left) and $2\bar{2}02$ (right).

**Figure 6:**
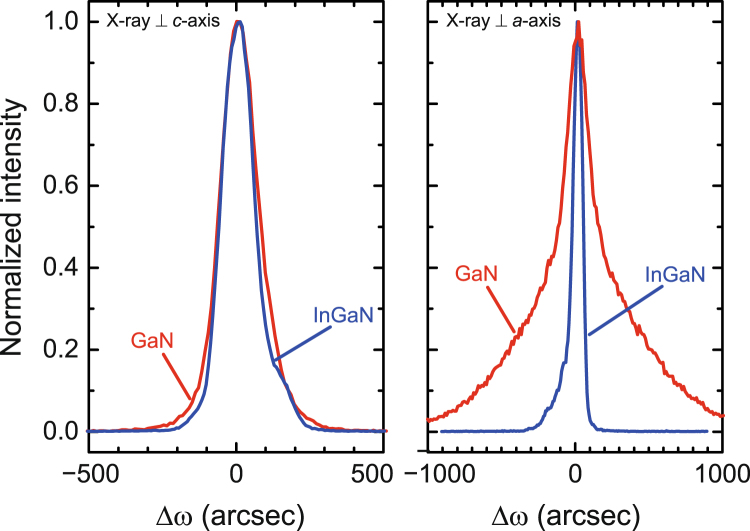
XRCs of $1\bar{1}00$ diffraction for *m*-plane GaN and InGaN grown on ZnO substrates.

Finally, the structural properties of the 180-nm-thick *m*-plane In_0.12_Ga_0.88_N grown on ZnO substrates were investigated. The film was grown at a temperature as low as 350 °C to prevent interfacial reactions between InGaN and ZnO. The RHEED and AFM analyses revealed that the *m*-plane InGaN grown on ZnO possesses atomically flat surfaces similar to those at of the *m*-plane GaN on ZnO. RSMs shown in Fig. 5 revealed that the growth of 180-nm-thick *m*-plane InGaN is coherent to the ZnO substrate, which supports the calculation shown in Fig. 4. The strains in In_0.12_Ga_0.88_N on ZnO were found to be +0.6% (*a*-axis), −0.8% (*c*-axis), and 0.0% (*m*-axis: normal to the surface). In this film, the strains along the *a*- and *c*-axes cancelled each other out, and no strains were applied along the *m*-axis. Figure 6 compares the XRCs for *m*-plane GaN and In_0.12_Ga_0.88_N grown at 350 °C. The thickness of both films was the same (180 nm). Since the 180-nm-thick GaN film released lattice strains by introducing structural defects, the FWHM of the rocking curves was as large as 288 arcsec. On the other hand, coherently grown *m*-plane InGaN exhibits a sharp rocking curve with an FWHM of 79 arcsec. These results indicate that the control of the lattice constant of InGaN by tuning the In composition is quite effective for obtaining high-quality nitride films on ZnO substrates. Electrical and optical properties of the InGaN films grown on ZnO are helpful information to assess the quality of the films. We tried to perform Hall-effect measurements of the InGaN films, but a reliable data was not obtained because of the conductive n-type ZnO substrate. In addition, for photoluminescence (PL) measurements using 325 nm excitation laser, ZnO emitted a strong UV luminescence (~380 nm), making it difficult to detect the PL coming from the thin InGaN film. If the InGaN film is peeled off from the substrate and transferred onto another substrate, the measurements would be successful. Unfortunately, we do not have a technique that enables the transfer of the thin InGaN film. The electrical and optical characterization of the film will be a next work.

The structural properties of *m*-plane GaN and InGaN films grown on lattice-matched ZnO substrates by PSD were investigated. It was found that *m*-plane GaN and InGaN films can be coherently grown at ~350 °C. Theoretical calculations have indicated that the critical thickness of *m*-plane In_0.12_Ga_0.88_N grown on ZnO is larger than that of *m*-plane GaN grown on ZnO, in agreement with the experimental results. The XRCs of *m*-plane In_0.12_Ga_0.88_N on ZnO were narrower than those of *m*-plane GaN on ZnO. This work is the first comprehensive study of the strains of nonpolar InGaN films on ZnO and it demonstrated the feasibility of ZnO as a substrate for growing nonpolar InGaN films. These nonpolar materials could be useful for fabricating long-wavelength (yellow and red) light-emitting devices.

## Methods

GaN and InGaN films were grown by PSD. High-purity Ga and In targets were separately sputtered in a Ar–N_2_ mixture gas. Before growing the film, the *m*-plane ZnO substrates were annealed in a ceramic ZnO box at 1025 °C^29^ so that the surface of substrate attained atomically flat steps. The growth temperature ranged from 300 °C to 540 °C. Structural characterization of the films was performed using RHEED, AFM, and XRD.
